# Detection of Ferric Ions and Catecholamine Neurotransmitters via Highly Fluorescent Heteroatom Co-Doped Carbon Dots

**DOI:** 10.3390/s20123470

**Published:** 2020-06-19

**Authors:** Thi Hoa Le, Hyun Jong Lee, Ji Hyeon Kim, Sang Joon Park

**Affiliations:** Department of Chemical and Biological Engineering, Gachon University, Seongnam 13120, Korea; hoale2907@gmail.com (T.H.L.); hjlee2@gachon.ac.kr (H.J.L.); jihyeon@gachon.ac.kr (J.H.K.)

**Keywords:** off-on fluorescence detector, bi-sensor, co-doped carbon dots, improved quantum yield

## Abstract

Carbon dots (CDs) demonstrate very poor fluorescence quantum yield (QY). In this study, with the help of a hydrothermal method, we combined CDs with nitrogen and phosphorus elements belonging to the VA group (in the periodic table) to form heteroatom co-doped CDs, i.e., nitrogen and phosphorus co-doped carbon dots (NPCDs). These displayed a significant improvement in the QY (up to 84%), which was as much as four times than that of CDs synthesized by the same method. The as-prepared NPCDs could be used as an “off-on” fluorescence detector for the rapid and effective sensing of ferric ions (Fe^3+^) and catecholamine neurotransmitters (CNs) such as dopamine (DA), adrenaline (AD), and noradrenaline (NAD). The fluorescence of NPCDs was “turned off” and the emission wavelength was slightly red-shifted upon increasing the Fe^3+^ concentration. However, when CNs were incorporated, the fluorescence of NPCDs was recovered in a short response time; this indicated that CN concentration could be monitored, relying on enhancing the fluorescence signal of NPCDs. As a result, NPCDs are considered as a potential fluorescent bi-sensor for Fe^3+^ and CN detection. Particularly, in this research, we selected DA as the representative neurotransmitter of the CN group along with Fe^3+^ to study the sensing system based on NPCDs. The results exhibited good linear ranges with a limit of detection (LOD) of 0.2 and 0.1 µM for Fe^3+^ and DA, respectively.

## 1. Introduction

Carbon dot (CD) is a new class of fluorescence carbon material that has been attracting considerable interest owing to their outstanding properties such as good water solubility, low toxicity, favorable biocompatibility, good photo-stability, tunable surface functionalities, and cell membrane permeability [1,2]. However, CDs have low quantum yields (QY) and few active sites, which limits their wider applications in fluorescent bioimaging [3,4,5], biosensing [6,7], ions and molecules detection [8,9], novel photoscience [10,11,12,13], and electrocatalysis [14]. Therefore, developing the methods to improve the fluorescent properties of CDs is extremely important. Previous studies proved that heteroatom doping is an effective and smart strategy to enhance their fluorescent performance [15]. Heteroatom doping provides available sites for target sensing [16,17] and can effectively control the band gap, electron density, leading to the QY enhancement of the CDs [18]. In addition, CDs doped with heteroatoms present distinctive photoluminescence (PL) behavior for application in various fields [19,20]. It is known that because nitrogen possesses a similar atomic size to carbon and has five strong valence bonds, it is considered to be a prominent chemical doping element for tailoring the electronic properties of CDs and can also bond strongly with the carbon atoms [21,22]. After doping with N atoms, CDs become n-type semiconductors. In the case where CDs act as n-type semiconductors, they may form substitution defects in sp^3^-bonded diamond thin films [23]. Hence, nitrogen and phosphorus co-doping would change the electronic characteristics of CDs, as well as control their physical and chemical properties, leading to higher QY.

Ferric ions (Fe^3+^) play vital roles in several biochemical and biophysical processes including DNA replication, cell proliferation, oxygen transport, enzyme catalysis, etc [24]. Consequently, the deficiency or excess of Fe^3+^ leads to various diseases. A disproportion in the number of Fe^3+^ may result in liver and kidney damage, megaloblastic, hemolytic anemia, and heart failure [25,26]. Moreover, determining Fe^3+^ levels is an important factor for evaluating the quality of drinking water [27]. Hence, detecting Fe^3+^ is extremely important in environmental and biomedical domains.

Similarly, catecholamine neurotransmitters (CNs) such as dopamine (DA), adrenaline (AD), and noradrenaline (NAD) play roles as messengers of neurological information [28]. Abnormal levels of CNs are related to various diseases including Parkinson’s, Alzheimer’s, schizophrenia, Huntington’s chorea, severe head trauma, neuroblastoma, pituitary adenoma, adrenocortical carcinoma, pheochromocytoma, various cancerous tumors, and depression [29,30]. Therefore, the growth of a selective and sensitive technique for detecting CN is crucial.

**Scheme 1:**
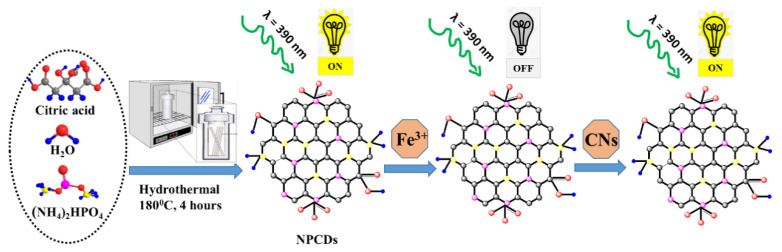
Illustration of NPCDs bi-sensor detecting Fe^3+^ and CNs based on the “off-on”mechanism.

In this study, we used a hydrothermal approach to prepare nitrogen and phosphorus co-doped CDs with excellent fluorescent properties with independent emission behavior and stability over a wide pH range. Then, the as-prepared NPCDs were used in simple and rapid fluorescent bi-sensor for detecting both Fe^3+^ and CNs (in particular, we chose DA as the representative of CN group), which is based on an “off-on” fluorescence mechanism (Scheme 1). First, the fluorescence intensity of NPCDs was quenched in the presence of Fe^3+^. Then by adding CNs to the solution, the intensity of the NPCDs/Fe^3+^ probe was improved. In fact, CNs can reduce Fe^3+^ to Fe^2+^, which cannot cause the fluorescence quenching effect. Besides, the catechol group of CNs can interact with Fe^3+^ and then remove Fe^3+^ from the surface of NPCDs [31,32], therefore, the quenched fluorescence is recovered. It is clear to see that the fluorescent bi-sensor opens a new potential technique for the detection of Fe^3+^ and CNs because of its easy preparation, simple-operation, high efficiency, and in-expensive cost when compared with other sensing methods, which require expensive instruments and complex operating processes [28,33].

## 2. Experimental Details

### 2.1. Materials

All chemicals were purchased from Sigma–Aldrich and were used without further purification. Chemicals consist of citric acid monohydrate, (NH_4_)_2_HPO_4_, FeCl_3_, DA, AD, NAD, glucose, fructose, H_2_O_2_, urea, glycine, alanine, and phosphate buffer saline (PBS) (pH = 7.4). Deionized (DI) water was utilized in all the processes.

### 2.2. Instruments and Measurements

Transmission electron microscopy (TEM) was conducted using TEM (FEI, Tecnai, F30S-Twin). The UV-Vis spectra and photoluminescence (PL) were obtained using a G1103A UV-Vis spectrophotometer from Agilent, USA and a QuantaMaster TM 50 PTI spectrofluorometer from Photon Technology International, USA. QY was conducted on FLUORO Q2100. Zeta potential measurements were measured by electrophoretic light scattering (Photal Otsuka Electronics, ELS 8000). X-ray photoelectron spectroscope (XPS) spectra were obtained with an X-ray photoelectron spectrometer (PHI 5000, Japan). Fourier transform infrared (FTIR) spectroscopy measurements were carried out using a Nicolet 6700 spectrometer from Thermo Scientific.

### 2.3. Synthesis of NPCDs

A hydrothermal method was used to synthesize the NPCDs. In particular, 2 g citric acid monohydrate and 5 g (NH_4_)_2_HPO_4_ (molar ratio of 1:4) were mixed with 30 mL water. Next, the mixture was transferred into a Teflon-lined stainless steel autoclave with a capacity of 50 mL. Subsequently, the mixture was heated at 180 °C for 4 h. After cooling the autoclave to room temperature, a dark brown colored solution was obtained. First, this product solution was filtered through a 0.22 µm polyethersulfone membrane, then further dialyzed using a dialysis bag (MWCO: 1000 Da). After 48 h of dialyzing, the obtained light violet-colored solution was finally lyophilized to acquire a powdery substance for later characterization. Undoped CDs were prepared following the above method without adding (NH_4_)_2_HPO_4_.

### 2.4. Quantum Yield Measurement 

The QY of NPCDs was measured by a comparative method using quinine sulfate in 0.1M H_2_SO_4_ as the standard (std) solution (QY_std_ = 0.54 at excitation wavelength of 360 nm, n_std_ = 1.33). The QY of NPCDs was calculated by Equation (1):

(1) $$Q_{NPCDs}=Q_{std}\frac{I_{NPCDs}\cdot A_{std}\cdot {\left(n_{NPCDs}\right)}^{2}}{I_{std}\cdot A_{NPCDs}\cdot {\left(n_{std}\right)}^{2}}$$

where Q, I, A, and n represent fluorescence quantum yield, integrated emission intensity, the refractive index of solvent, and absorbance, which is measured by UV-Vis spectrophotometer, respectively. 

### 2.5. Procedure for Detecting Fe^3+^

The Fe^3+^ detecting process was conducted at room temperature. First, 60 µL of NPCDs were added to 3 mL DI water. Next, varying volumes of Fe^3+^ solution were dropped into the NPCDs solution to obtain different concentrations (0, 0.2, 0.5, 1, 3, 5, 10, 15, 20, 25, 30, 40, and 50 µM). The solution was further diluted to the mark using DI water and then was equilibrated for 1 min before fluorescence measurement under an excitation wavelength of 390 nm. This testing for each concentration was repeated three times.

### 2.6. Diagnosis of CNs

As mentioned in the Introduction, we choose DA as the representative of the CN group for investigation. In a specific test, 60 µL NPCDs were dropped into 3 mL of PBS (pH = 7.4) first. In order to form an NPCDs/Fe^3+^ probe, a fixed amount of Fe^3+^ was added to the solution. Then, to study the sensitivity of the NPCDs/Fe^3+^ probe towards DA, varying concentrations of DA (0, 2, 5, 10, 20, 50, 70, 90, 100, 120, 150, 200, 250, and 300 µM) were adjusted by adding different amount of DA solution to the mixture. The solution was further diluted to the point with DI water. After 10 min of incubation at room temperature, the fluorescence restore spectra were measured with an excitation wavelength of 390 nm. Measurements were taken in triplicate for each concentration.

In addition, to determine the selectivity of the NPCDs/Fe^3+^ probe towards DA, the effect and interferences of other reducing molecules were investigated.

## 3. Results and Discussions

### Characterization of the NPCDs

Fluorescence and UV-Vis absorption spectroscopy were used to characterize the optical properties of NPCDs. The UV-Vis spectrum (Figure S1) shows that a small peak appears at 234 nm that lies next to a strong peak at 334 nm, which originated owing to trapped excited-state energy from surface states [1,18,34].

**Figure 1:**
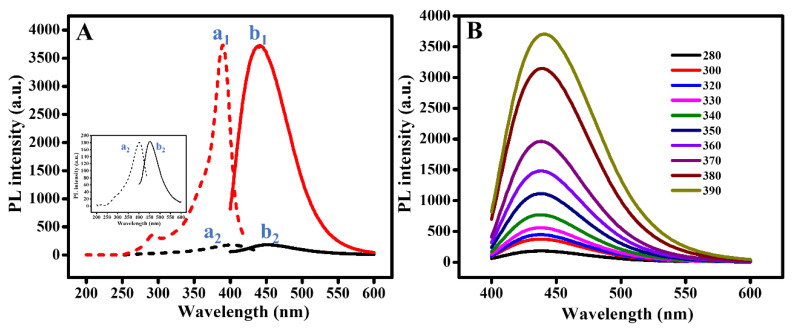
(**A**) Excitation (a_1_, a_2_) and emission (b_1_, b_2_) fluorescence spectra of the CDs and NPCDs (inset: enlarge fluorescence spectrum of CDs), (**B**) fluorescence spectra of the NPCDs in the range of excitation wavelength from 280 to 390 nm.

It can be seen from Figure 1A, the CDs fluorescence emission reached the maximum peak at 452 nm under an excitation wavelength of 402 nm, whereas the maximum fluorescence emission peak of the NPCDs was observed at a shorter wavelength of 440 nm when the excitation wavelength was 390 nm. In addition to the change in the location of the emission peaks, the fluorescence intensity of the NPCDs was sharply increased compared to that of the CDs alone. Moreover, NPCDs exhibited a QY of 84% that was four times greater than that of undoped CDs. In the Introduction, we explained the reason why N and P doping can enhance the fluorescence properties of CDs. Table 1 shows the QY comparison of our NPCDs with NPCDs prepared using different methods and CDs co-doped with other elements. It is clear to see that the NPCDs synthesized by our method have an excellent QY when compared to the others.

In addition, we investigated excitation-dependent fluorescence emissions of CDs and NPCDs solutions. As for CDs, when the value of the excitation wavelength was increased, the fluorescence intensity went up along with a red-shift (Figure S2). As for the NPCDs, Figure 1B shows that there was an enhancement in fluorescence intensity; however, there was no shift of emission peaks in the range of excitation wavelengths from 280 to 390 nm. The excitation-independence of the NPCDs indicates that the NPCDs had narrow size distributions and homogeneous surface structures; this was further confirmed using TEM results.

**Figure 2:**
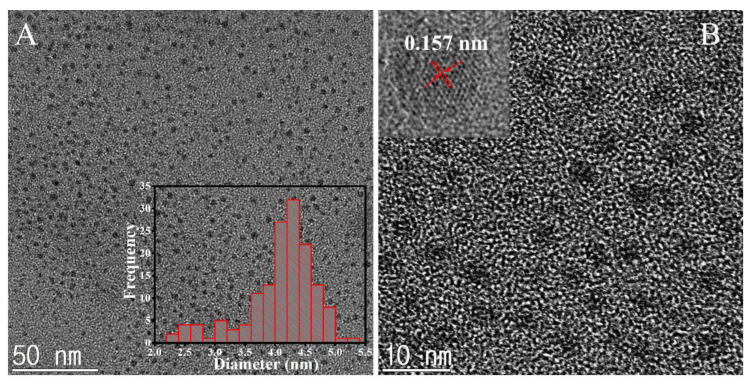
TEM images of NPCDs (inset (**A**) size distribution (**B**) HRTEM image of NPCDs).

The morphologies of the NPCDs are displayed via TEM images. Moreover, particle size statistics were calculated by measuring 151 particles and the result displayed a narrow size distribution ranging from 2.3 to 5.3 nm. Monodisperse NPCDs were synthesized with an average size of 4.1 nm (standard deviation of 0.6 nm). In the high-resolution (HR) TEM image (inset of Figure 2B), the crystal structure of the CDs was visible and the lattice parameter was 0.157 nm, which was associated with graphitic diffraction planes [39,40].

**Figure 3:**
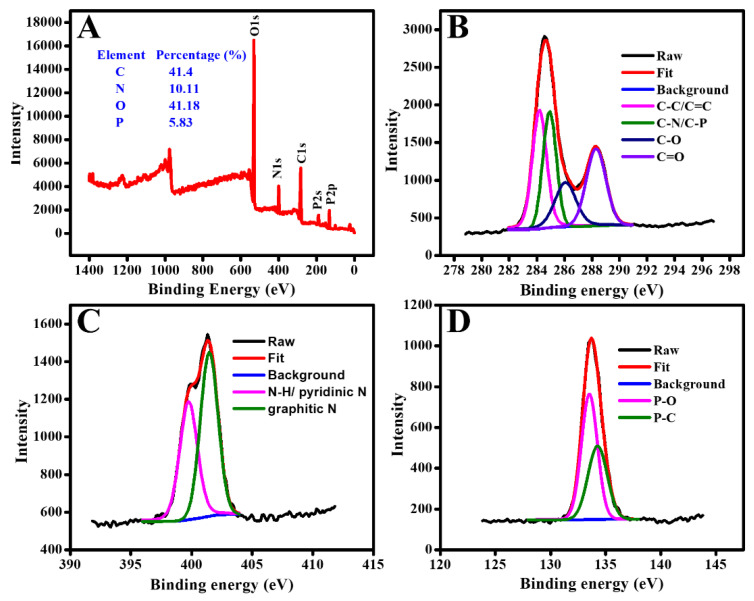
(**A**) XPS survey spectra of NPCDs. High-resolution XPS spectra of (**B**) C1s, (**C**) N1s, and (**D**) P2p, respectively.

The XPS spectrum (Figure 3A) presents five typical peaks at 280.5, 399.5, 529.5, 187.6, and 132.3 eV, corresponding to C1s, N1s, O1s, P2s, and P2p, respectively. The atomic percentages of C, N, O, and P were analyzed to be 41.4%, 10.11%, 41.18%, and 5.83%, respectively, which confirmed that the CDs were incorporated with N and P. In Figure 3B, C1s peak can be parsed to four peaks placed at 284.2, 285, 286, and 288.4 eV, being suitable for C–C/C=C, C–N/C–P, C–O, and C=O bonds, respectively, originating from the sp^2^ graphitic structure [35] and several carboxyl, hydroxyl, amine, phosphate, etc. groups on the surface of the NPCDs; this can be further confirmed from the P2p and N1s spectra. The P2p spectrum (Figure 3D) presents two characteristic peaks, corresponding to P–O at 133.5 eV and P–C at 133.8 eV. With reference to the N1s spectrum (Figure 3C), N1s peak can be de-convoluted to a peak position at 399 eV in accord with the N–H bond of an amine group or pyridinic N and one located at 401.5 eV assigned to graphitic N [41,42].

**Figure 4:**
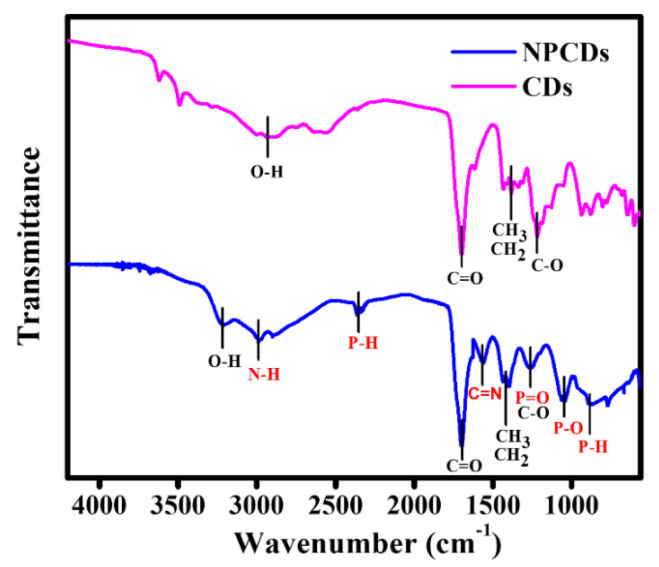
FTIR spectra of CDs and NPCDs.

Moreover, we characterized CDs and NPCDs by FTIR spectra. It can be seen from Figure 4 that the spectrum of CDs shows main characteristic peaks, including a broad absorption band at 3300 to 2500 cm^−1^ that owe to O–H stretching of the carboxylic and hydroxyl groups; peaks at 1702 and 1211 cm^−1^, that result from the C=O bonding and C–O vibrations, respectively; and peaks at 1379 cm^−1^, which is due to the bending vibrations of the CH_2_ and CH_3_ groups. The spectrum of the NPCDs not only includes all the peaks belonging to the CDs, but further contains specific peaks indicating the existence of N and P elements. A broad peak in the range of 3000 to 2800 cm^−1^ is a consequence of N–H stretching whereas the peak at 1600 cm^−1^ is caused by C=N stretching vibration. The peaks appearing at 2332 cm^−1^ and 874 cm^−1^ are results of P–H stretching and bending, respectively. Moreover, P=O and P–O stretching vibrations lead to the appearance of peaks at 1259 cm^−1^ and 1047 cm^−1^, respectively. Consequently, the XPS result and FTIR spectra are consistent, confirming that N and P elements were successfully co-doped into the CDs.

## 4. Bi-Sensing of Fe^3+^ and CNs

### 4.1. Fe^3+^ Detection Based on NPCDs

**Figure 5:**
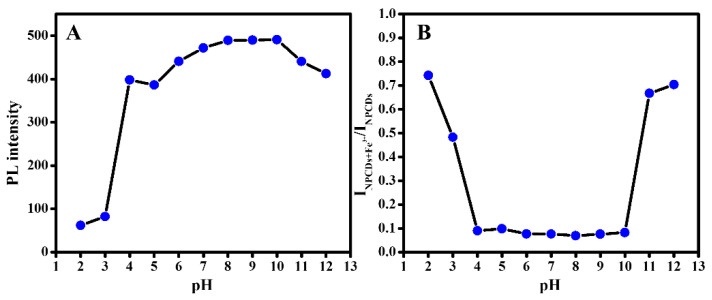
(**A**) Fluorescence intensity of NPCDs and (**B**) quenching effect of Fe^3+^ on the fluorescence of NPCDs in a variety of pH values.

First, we investigated the pH influence that is important to NPCDs fluorescence intensity as well as NPCDs fluorescence response to Fe^3+^. Figure 5A displayed a very low fluorescence intensity of NPCDs in strongly acidic solutions. It reached to the maximum and kept stable in the range of pH from 7 to 10. In the presence of Fe^3+^ (Figure 5B), the quenching effect on NPCDs fluorescence intensity occurred strongly when the pH changed from 4 to 10. As for strongly alkaline conditions, Fe^3+^ ions are easily precipitated owing to the formation of a Fe–OH complex [43], which may affect the quenching phenomenon. From these results, we realized that a value of pH belonging in the range of 7 to 10 is suitable for Fe^3+^ detection. In particular, we chose PBS buffer (pH = 7.4) for this Fe^3+^ sensing.

**Figure 6:**
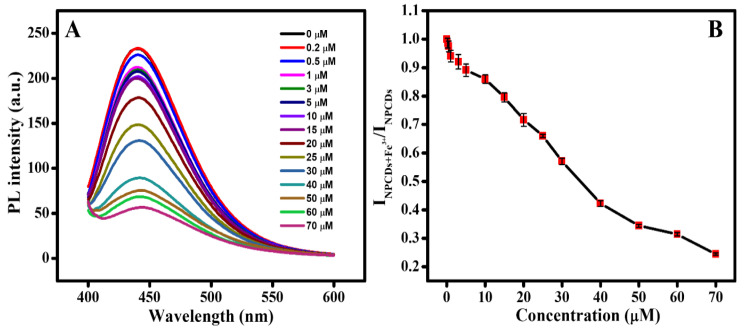
(**A**) Fluorescence spectra of NPCDs in the presence of different concentrations of Fe^3+^ and (**B**) the linear relationship between *I_NPCDs+Fe^3+^_*/*I*_NPCDs_ and the concentration of Fe^3+.^

Figure 6 clearly shows the quenching impact of Fe^3+^ on the fluorescence intensity of the NPCDs. The NPCDs fluorescence intensity went down steadily along with a very slight red-shift corresponding to the increase in Fe^3+^ concentration from 0.2 to 70 µM (Figure 6A). When the level of Fe^3+^ was within the range of 0 to 40 µM, the calibration curve between the relative NPCD fluorescence intensity and Fe^3+^ concentration displayed a good linear relationship with the quenching equation, i.e., I_NPCDs+Fe_^3+^//I_NPCDs_ = 0.97994–0.01351 CM(Fe^3+^) and a correlation coefficient (R^2^) value of 0.99058. The experiment was repeated three times and data were expressed as the near ± standard deviation. Moreover, the LOD was determined to be approximately 0.2 µM, which is both considerably lower than the Fe^3+^ limitation amount in drinking water (5.4 µM) [44] and other previous methods [15,27,45]. Table 2 shows the comparison of our fluorescence probe results with others that have been developed for Fe^3+^ detection. It is clear that the present method provided excellent results for linear range and LOD. In addition, when investigating fluorescence responses of the NPCD probe to different metal cations, the NPCDs show high selectivity to Fe^3+^ (Figure S3).

### 4.2. NPCDs/Fe^3+^ as Fluorescence Probe for CN Detection

**Figure 7:**
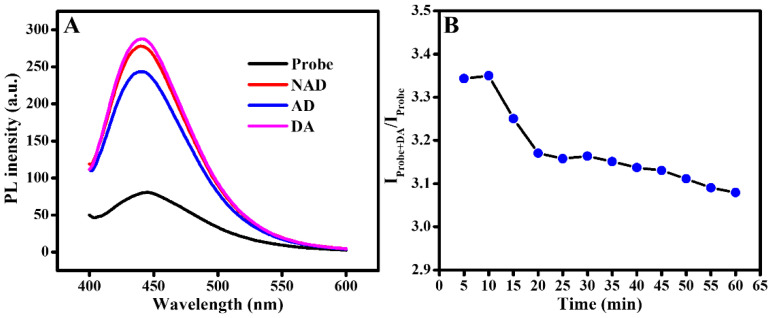
(**A**) Fluorescence intensity of the NPCDs/Fe^3+^ probe in the existence of various CNs at the same concentration of 5 µM, (**B**) time-dependence of fluorescence ratio (I_Probe+DA_/I_Probe_) in the presence of 5 µM DA.

Figure 7A clearly shows that the fluorescence intensity of NPCDs/Fe^3+^ was recovered dramatically after a certain incubation period on adding CNs such as DA, AD, and NAD with the same concentrations of 5 µM. This confirms that we can use this fluorescence “turn on” phenomenon for the sensing of CNs. In particular, we chose DA—the most important neurotransmitter of the CN group as a representative to investigate further. 

As mentioned above, NPCDs keep high fluorescence intensity in solutions with a pH of 7 to 10. In addition, according to previous reports, in alkaline solutions, adherent polydopamine will be produced through the self-polymerization process of DA [51,52], which is a drawback in the accurate detection of DA. Therefore, we used PBS (pH = 7.4) as a medium for DA determination. Moreover, time is an important factor affecting the fluorescence responses. After studying the fluorescence enhancement of NPCDs/Fe^3+^ probe towards DA by controlling time-dependent variations (Figure 7B), we observed that the optimized time required for detection is 10 min. The short time of incubation is a remarkable feature of this method.

**Figure 8:**
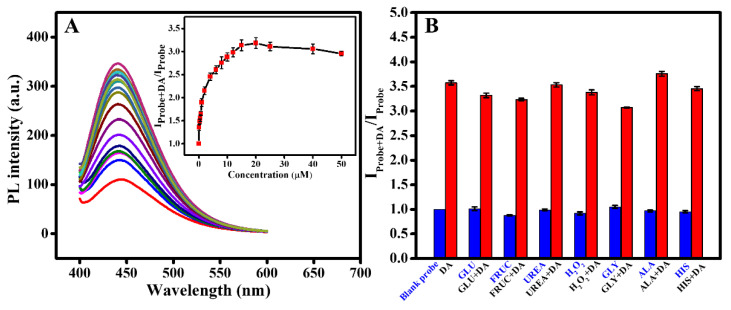
(**A**) Enhancement effect of DA on fluorescence intensity of NPCDs/Fe^3+^ probe (inset: calibration curve between I_Probe+DA_/I_Probe_ and concentration of DA), (**B**) selectivity of NPCDs/Fe^3+^ probe towards different interferences in the presence and absence of DA in an equal concentration of 5 µM.

Figure 8A shows that fluorescence of the NPCDs/Fe^3+^ gradually increased in the existence of varying concentrations of DA from 0 to 20 µM. Thereafter, an increase in the level of DA did not enhance fluorescence intensity any further. Indeed, the fluorescence-enhanced efficiency showed a superb linear response to DA concentration from 4 to 15 µM with equation I_Probe+DA_/I_Probe_ = 2.22411 + 0.0632 C_M_ (DA) and R^2^ value of 0.9892. The experiment was repeated in triplicate and the data were expressed as mean ± standard deviations. A LOD of 0.1 µM is an acceptable value.

To verify the specificity of NPCDs/Fe^3+^ probe towards DA, the fluorescent responses of the probe in the presence of potentially interfering substances were investigated. As shown in Figure 8B, other analogs did not produce variations in the fluorescence intensity of NPCDs/Fe^3+^; however, on adding DA the fluorescence of the system enhanced significantly. It may be inferred that the enhancement effect did not occur with other substances and they did not have any effect on DA to turn on the fluorescence of the system, which showed that the NPCDs/Fe^3+^ probe exhibited excellent selectivity for CN detection.

### 4.3. “Off-On” Effect of Fe^3+^ and CNs on NPCDs Fluorescence

There are certain reasons for the quenching effect of Fe^3+^ on NPCDs fluorescence. First, Fe^3+^ may combine with amino and phosphoric acid groups belonging to the NPCDs surface, therefore, electrons may transfer from excited state S_1_ of the NPCDs to 3d half-filled orbits of Fe^3+^, result in quenching in fluorescence intensity [53]. This is confirmed by measuring the zeta potential value. After adding Fe^3+^, the zeta potential of NPCDs solution varied from −15.92 mV to −5.75 mV. Hence, there was an electrostatic interaction (electron exchange) between NPCDs and Fe^3+^. This was further supported by the dependence of Fe^3+^ detecting the performance of NPCDs over a wide pH range. Secondly, Fe^3+^ may coordinate with substantial phenol (Ar-OH) groups of NPCDs to form Fe–OAr complexes that work as absorbers to quench NPCD fluorescence and cause slightly red-shifted emission wavelengths [54,55]. Finally, there was a change in the average fluorescence lifetime of NPCDs from 2.47 ns (τ_1_ = 3.298 ns (74.33%), τ_2_ = 0.104 ns (25.67%)) to 1.86 ns (τ_1_ = 2.794 ns (63.72%), τ_2_ = 0.206 ns (36.28%)) in the existence of Fe^3+^; this confirmed that dynamic quenching occurred during this process [56].

**Scheme 2:**
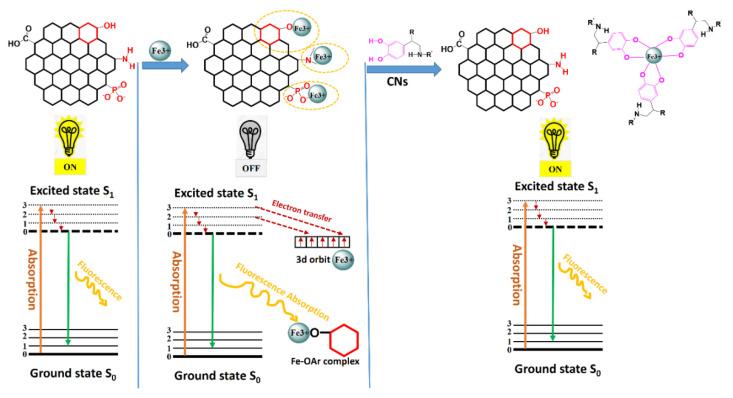
“Off-on” mechanism of Fe^3+^ and CNs on NPCDs fluorescence.

In the case of adding CNs to NPCDs/Fe^3+^ solution, there is an assumption that CNs can reduce Fe^3+^ to Fe^2+^ that cannot cause a fluorescence quenching effect leading to fluorophore formation. The main reason is that the catechol group of CNs can interact with Fe^3+^ and then remove Fe^3+^ from the surface of NPCDs [31,32] such that they can inhibit electron movement and fluorescence absorption process between them; therefore, the quenched fluorescence is recovered. This is the reason why, in particular when adding DA, the value of the zeta potential and average fluorescence lifetime was recovered to −12.05 mV and 2.094 ns (τ_1_ = 3.264 ns (63.05%), τ_2_ = 0.1001 ns (36.95%)), respectively. The mechanism is clearly described in Scheme 2.

## 5. Conclusions

We successfully synthesized nitrogen and phosphorus co-doped CDs comprising outstanding fluorescence properties compared to those of traditional undoped CDs. We applied NPCDs to design a bi-sensor for the detection of Fe^3+^ and CNs relying on an “off-on” mechanism. The fluorescence of NPCDs was quenched with an increase in Fe^3+^ concentration, and a linear range of 0 to 40 µM with a LOD value of 0.2 µM was obtained. After adding Fe^3+^, the NPCDs/Fe^3+^ system was formed, which acted as a fluorescence probe to determine CNs. The fluorescence of NPCDs/Fe^3+^ was recovered in the presence of CNs. We selected DA—the most important neurotransmitter of the CN group in our study—and the results displayed a superb linear range of 4 to 15 µM with an LOD value of 0.1 µM. With remarkable features such as simple design, speed, sensitivity, and selectivity, the sensor developed in this study establishes a strategy for successful application in the sensing of Fe^3+^ and CNs in real samples and furthers the development of simple, low cost, and sensitive sensors for potential applications in biomedical systems and environments.

## Figures and Tables

**Table 1:** Comparison of QY of co-doped CDs synthesized using different methods.

Doped Carbon Dots	Material	Method	Quantum Yield	Reference
N,P-CDs	Citric acid, O-phosphorylethanolamine	Hydrothermal	8.17%	[22]
N,P-CDs	Ethylenediamine, H_3_PO_4_	Hydrothermal	25.47%	[21]
N,P-CDs	Sodium citrate, (NH_4_)_2_HPO_4_	Hydrothermal	53.8%	[35]
N,P-CDs	Glucose, ammonia, H_3_PO_4_	Hydrothermal	54%	[24]
N,P-CDs	Citric acid, (NH_4_)_2_HPO_4_	Hydrothermal	84%	Our method
P,Cl-CDs	Maltose, H_3_PO_4_, HCl	Hydrothermal	15%	[18]
Si,N-CDs	Ethylenediamine, APTES	Hydrothermal	29.7%	[15]
S,N-CDs	Citric acid, N-acetyl-L-Cysteine	Hydrothermal	49%	[36]
S,N-CDs	Sodium citrate, sulfamide	Hydrothermal	55%	[37]
S,N-CDs	Citric acid, L-Cysteine	Hydrothermal	73%	[38]

**Table 2:** Comparison of different fluorescence probes for Fe^3+^ detection.

Materials	Linear Range (µM)	LOD (µM)	Reference
CDs	0−6.0	0.4	[46]
CDs	0−20	0.32	[47]
CDs	0−30	0.015	[48]
N-CDs	0−500	0.15	[49]
N,S-CDs	6−200	0.8	[50]
P,Cl-CDs	0.1−8.0	0.06	[18]
N,P-CDs	0−40	0.2	Our work

## Supplementary Materials

The following are available online at https://www.mdpi.com/1424-8220/20/12/3470/s1, Figure S1: UV-vis absorption spectrum of NPCDs; Figure S2: Fluorescence spectra of the CDs at different excitation; Figure S3: Selectivity of NPCDs for assaying Fe^3+^. All metal cations have same concentration of 50 μM.
