# Supplementary material for: Detection of Ferric Ions and Catecholamine Neurotransmitters via Highly Fluorescent Heteroatom Co-Doped Carbon Dots
Source: Sensors (Basel). 2020 Jun 19;20(12):3470. doi: 10.3390/s20123470 (PMC7349486; doi:10.3390/s20123470)
Supplement: Supplementary file 1 [file sensors-20-03470-s001.pdf]

# SUPPORTTING INFORMATION

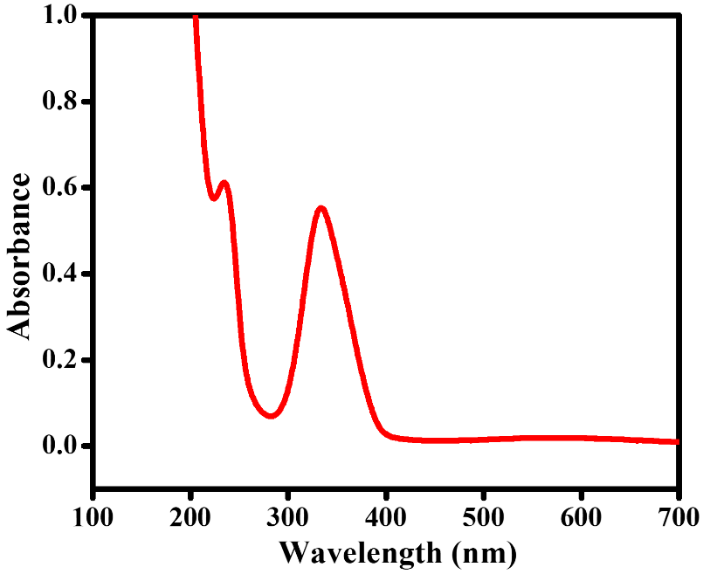


**Figure. S$_1$.** UV-vis absorption spectrum of NPCDs.

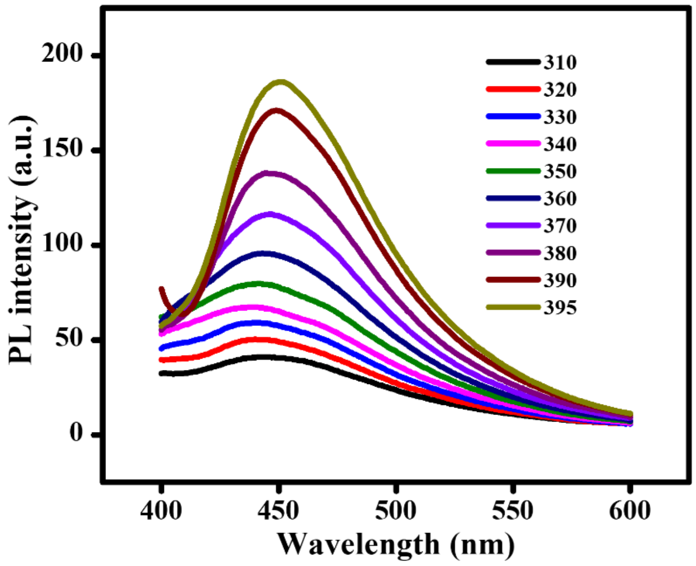


**Figure. S$_2$.** Fluorescence spectra of the CDs at different excitation.

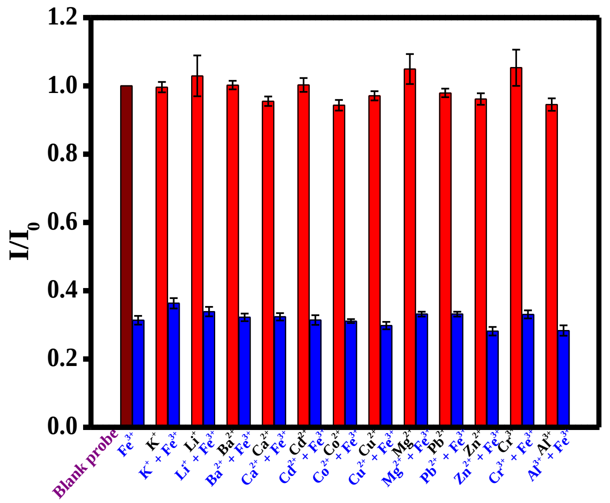


**Figure S$_3$:** Selectivity of NPCDs for assaying $Fe^{3+}$. All metal cations have same concentration of 50 μM.
